# T stage and venous invasion are crucial prognostic factors for long-term survival of patients with remnant gastric cancer: a cohort study

**DOI:** 10.1186/s12957-021-02400-5

**Published:** 2021-09-27

**Authors:** Kentaro Matsuo, Sang-Woong Lee, Ryo Tanaka, Yoshiro Imai, Kotaro Honda, Kohei Taniguchi, Hideki Tomiyama, Kazuhisa Uchiyama

**Affiliations:** 1 Department of General and Gastroenterological Surgery, Osaka Medical and Pharmaceutical University, 2-7 Daigaku-machi, Takatsuki, Osaka, 569-8686 Japan; 2 Translational Research Program, Osaka Medical and Pharmaceutical University, 2-7 Daigaku-machi, Takatsuki, Osaka, 569-8686 Japan

**Keywords:** Remnant gastric cancer, Long-term outcome, Clinicopathological factor, Prognosis

## Abstract

**Background:**

The incidence of remnant gastric cancer (RGC) after distal gastrectomy is 1–5%. However, as the survival rate of patients with gastric cancer improves due to early detection and treatment, more patients may develop RGC. There is no consensus on the surgical and postoperative management of RGC, and the clinicopathological characteristics correlated with the long-term outcomes remain unclear. Therefore, we investigated the clinicopathological factors associated with the long-term outcomes of RGC.

**Methods:**

We included 65 consecutive patients who underwent gastrectomy for RGC from January 2000 to December 2015 at the Osaka Medical and Pharmaceutical University Hospital, Japan. The Kaplan–Meier method was used to create survival curves, and differences in survival were compared between the groups (clinical factors, pathological factors, and surgical factors) using the log-rank test. Multivariate analyses using the Cox proportional hazard model were used to identify factors associated with long-term survival.

**Results:**

No significant differences were noted in the survival rate based on clinical factors (age, body mass index, diabetes mellitus, hypertension, cardiovascular disease, pulmonary complications, liver disease, diet, history of alcohol drinking, and history of smoking) or the type of remnant gastrectomy. Significant differences were noted in the survival rate based on pathological factors and surgical characteristics (intraoperative blood loss, operation time, and the number of positive lymph nodes). Multivariate analysis revealed that the T stage (hazard ratio, 5.593; 95% confidence interval [CI], 1.183–26.452; *p* = 0.030) and venous invasion (hazard ratio, 3.351; 95% CI, 1.030–10.903; *p* = 0.045) were significant independent risk factors for long-term survival in patients who underwent radical resection for RGC.

**Conclusions:**

T stage and venous invasion are important prognostic factors of long-term survival after remnant gastrectomy for RGC and may be keys to managing and identifying therapeutic strategies for improving prognosis in RGC.

**Supplementary Information:**

The online version contains supplementary material available at 10.1186/s12957-021-02400-5.

## Background

Remnant gastric cancer (RGC) describes all cancers arising from the remnant stomach after partial gastrectomy, regardless of the initial disease or type of gastrectomy [1]. The incidence of RGC after distal gastrectomy has been reported to be 1–5% [2–4]. Although the number of patients with RGC who undergo gastrectomy for benign diseases has decreased due to improvements in treatment, more patients with a previous malignant disease are developing RGC because of improved prognosis after gastric cancer [5].

The etiology of RGC is believed to be related to the type of reconstruction. For instance, the anastomotic site of Billroth II reconstruction, which is exposed by bile regurgitation, is a common site of recurrence [6, 7]. However, non-anastomotic carcinoma occurs more frequently in patients with previous malignancies who have undergone Billroth I reconstruction [8]. Some researchers have reported that the prognosis of advanced RGC is worse than that of primary advanced gastric cancer [9]. However, despite these findings, there has been no consensus on the surgical and postoperative management for RGC, and the clinicopathological characteristics that are correlated with long-term outcomes remain unclear. Data collection on the prognoses of patients with RGC is required to establish an optimal therapeutic strategy for RGC. Herein, we have investigated the clinicopathological factors associated with the long-term outcomes of RGC.

## Methods

### Patients

From January 2000 to December 2015, 65 consecutive patients with RGC underwent gastrectomy at the Osaka Medical College Hospital, Japan. We performed routine workup, including esophagogastroduodenoscopy (EGD) and enhanced computed tomography (CT), for preoperative evaluation. Retrieved specimens were staged using the Japanese Classification of Gastric Carcinoma (15th edition); the depth of tumor invasion was recorded as the pathological T stage. Tumor morphology was categorized as either superficial (pT1) or advanced (pT2–pT4). Lymph node metastasis was defined using the pathological N category, and lymphatic invasion and venous invasion were also assessed.

Clinical, surgical, and pathological records of the patients were obtained from our database. Data collection (after receiving written informed consent) and analysis were approved by the Institutional Review Board of the Osaka Medical College (acceptance number: 2020–005). Written informed consent was obtained from all participants.

### Patient follow-up

After surgery, blood tests and physical examinations were performed every 3 months, CT was performed every 6 months, and EGD was performed annually. The blood tests also included examination of tumor markers, such as the carcinoembryonic antigen and carbohydrate antigen 19–9. Postoperative adjuvant chemotherapy was not administered to most patients.

The duration of follow-up was 60 months. Thirty-six patients were completely followed up, and 29 were lost to follow-up due to disease-specific death (*n* = 19), death from other causes (*n* = 3), and unknown reasons (*n* = 7).

### Statistics

All statistical analyses were performed using JMP version 15.0 software (SAS Institute Inc., Cary, NC, USA). The Kaplan–Meier method was used to estimate survival curves, and differences in survival were compared using the log-rank test. The cutoff value was set for each factor (age, body mass index [BMI], tumor size, blood loss, operation time, and the number of retrieved lymph nodes and positive lymph nodes) by using a receiver operating characteristic curve analysis.

The multivariate analysis was performed using the Cox proportional hazard models, and *p* < 0.05 was considered significant.

## Results

### Patient characteristics

The clinicopathological and surgical characteristics of the 65 patients are summarized in Tables 1 and 2. The patients comprised 55 men and 10 women. The median age was 71 years (interquartile range, 63.5–76 years). Forty-eight patients (74%) originally had a malignant disease, and 17 (26%) had a benign disease. The median interval between original gastrectomy and development of RGC was 10 years (interquartile range, 4.25–15 years) in patients who originally had a malignant disease, and 30 years (interquartile range, 24.5–42 years) in patients who originally had a benign disease. Total resection of the remnant stomach was performed in 49 patients, while partial resection was performed in 16 patients.

**Table 1:** Demographic and clinical characteristics of the patients undergoing remnant gastrectomy

Characteristics	Patients
	(*n =* 65)
Age, years	
Median	71
Interquartile range	63.5–76
Sex	
Male	55 (84.6%)
Female	10 (15.4%)
ASA	
1	8 (12.3%)
2	50 (76.9%)
3	7 (10.8%)
Body mass index, kg/m^2^	
Median	20.8
Interquartile range	19.2–23.4
Previous disease	
Benign	17
Malignant	48
Previous reconstruction	
Billroth I	23 (35.4%)
Billroth II	20 (30.8%)
Others	22 (33.8%)
Years since previous surgery	
Median	12
Interquartile range	6.5–24.5
No. of comorbidities	
0	24 (36.4%)
1–2	37 (56.1%)
≥3	5 (7.5%)

*ASA*, American Society of Anesthesiologists

**Table 2:** Demographic, pathological, and surgical characteristics of the patients undergoing remnant gastrectomy

Characteristic	Patients
	(*n =* 65)
Histologic type	
Differentiated	58 (89.2%)
Undifferentiated	7 (10.8%)
Pathological T factor	
1/2/3/4	30/7/16/12
Pathological N factor	
0/1/2/3/X	43/9/8/1/4
Pathological Stage	
I/II/III/IV	37/10/9/7
Type of remnant gastrectomy	
Total	49 (73.2%)
Partial	16 (26.8%)
Operation time, min	
Median	320
Interquartile range	260–372.5
Blood loss, mL	
Median	310
Interquartile range	209–515
Number of retrieved lymph nodes	
Median	12
Interquartile range	6–19
Number of positive lymph nodes	
Median	0
Interquartile range	0–1
0	43
1–3	10
4–20	8
X	4
Adjuvant therapy	
Yes	4 (6.1%)
No	61 (93.9%)

### Association of long-term survival with clinical, pathological, and surgical factors

**Fig. 1:**
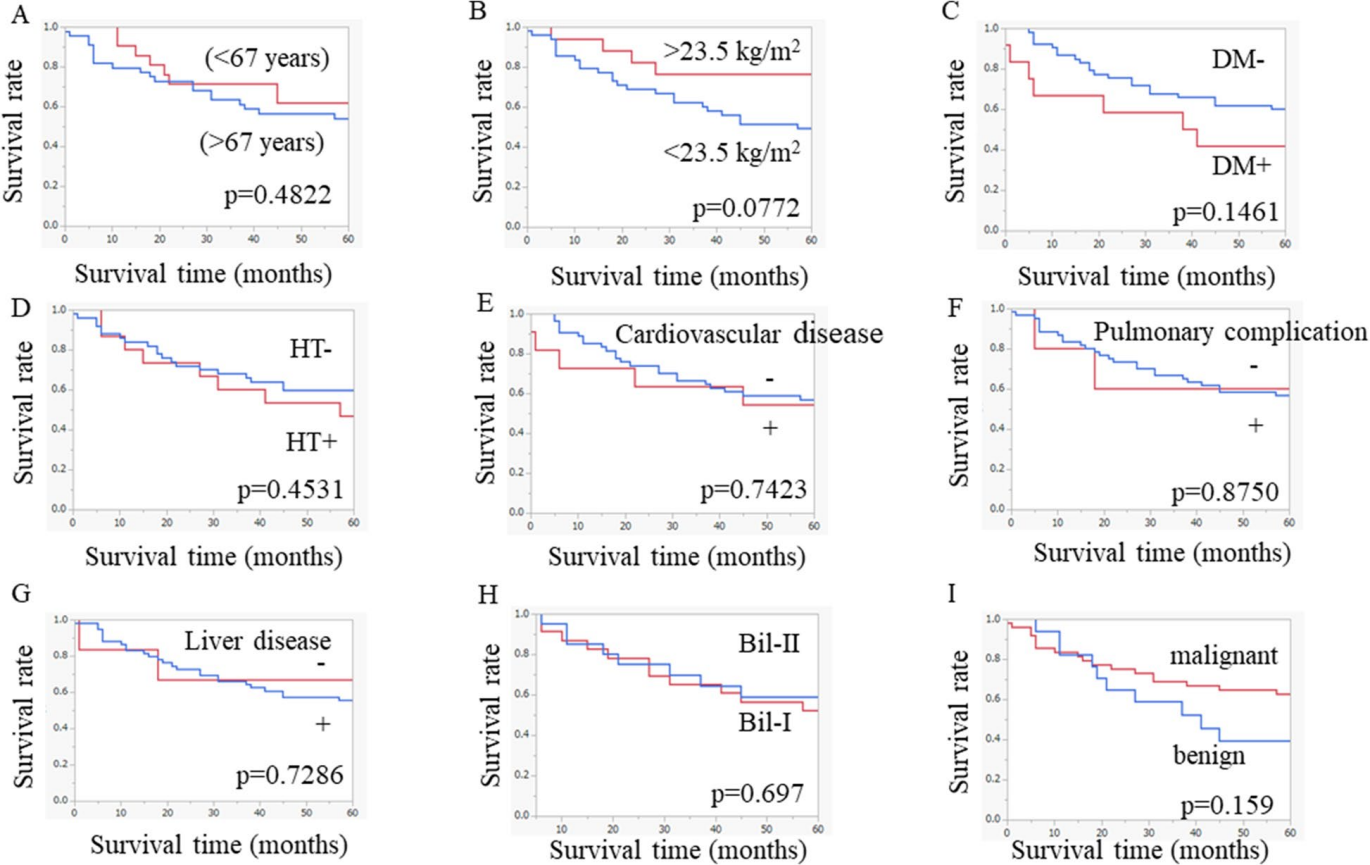
Long-term survival outcomes based on clinical factors: **A** age, **B** body mass index, **C** diabetes mellitus (DM), **D** hypertension (HT), **E** cardiovascular disease, **F** pulmonary complications, **G** liver disease, **H** type of reconstruction, and **I** previous disease

The overall survival curves based on the clinical characteristics are shown in Fig. 1. There were no significant differences in the survival between patients with values above and below the cut-offs for any of the clinical factors that were examined, including age (older [> 67 years] vs. younger [< 67 years]), BMI, diabetes mellitus, hypertension, cardiovascular disease, pulmonary complications, liver disease, type of original reconstruction, or previous disease history. In addition, overall survival curves based on diet, history of alcohol drinking, and history of smoking are shown in Supplemental Fig. 1.

**Fig. 2:**
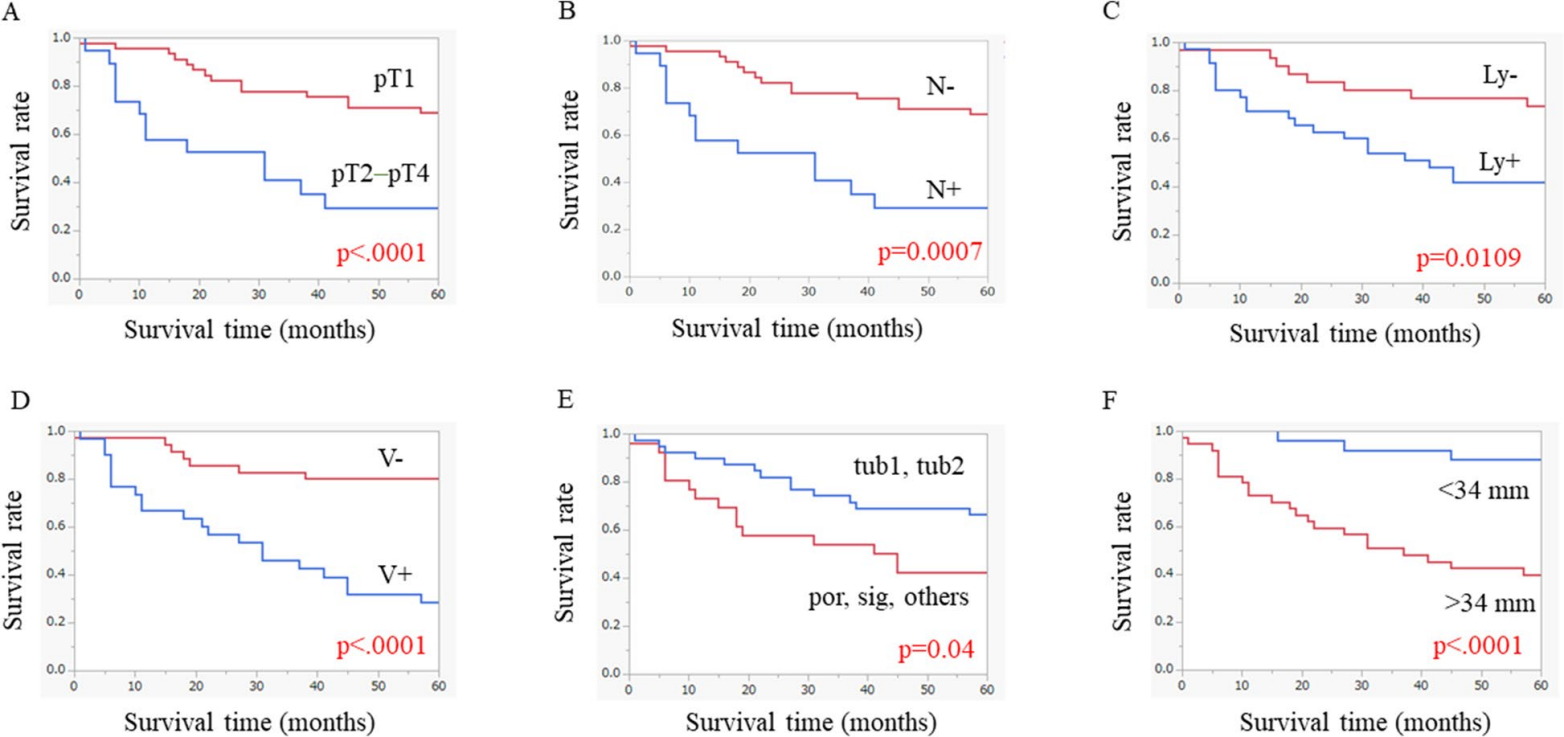
Long-term survival outcomes based on pathological factors: **A** pathological T stage, **B** lymph node metastasis, **C** lymphatic invasion, **D** venous invasion, **E** histological type, and **F** tumor size

The overall survival curves based on the pathological characteristics are shown in Fig. 2. There were significant differences in the survival rate between patients with values above and below the cut-offs for all pathological factors examined.

The overall survival curves based on each pT stage are shown in Supplemental Fig. 2. There were significant differences in the survival between patients with tumors of different pathological T stages (pT1 and pT2, pT1 and pT3, and pT1 and pT4).

**Fig. 3:**
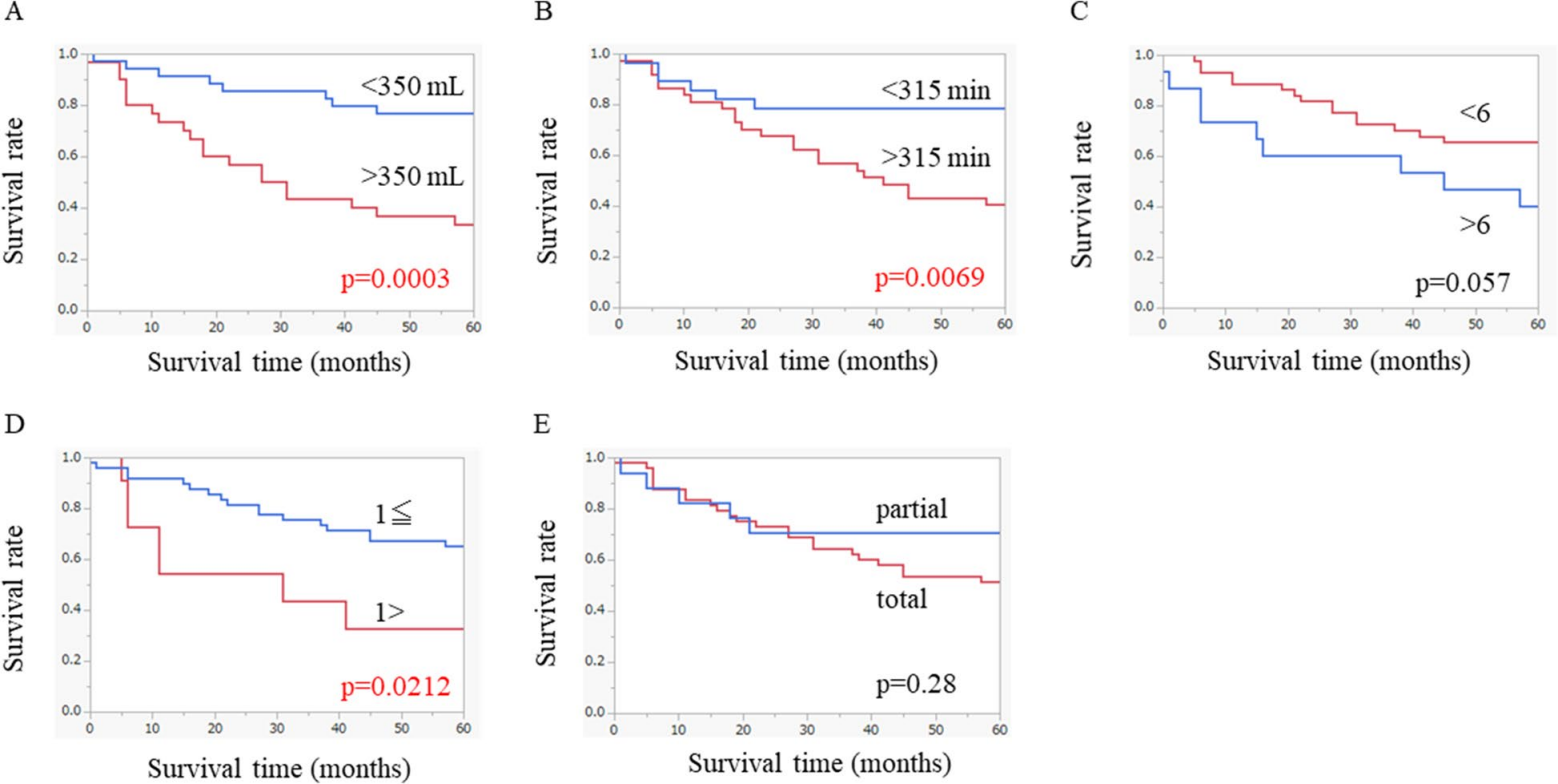
Long-term survival outcomes based on surgical factors: **A** intraoperative blood loss, **B** operation time, **C** number of retrieved lymph nodes, **D** number of positive lymph nodes, and **E** type of remnant gastrectomy

The overall survival curves based on the surgical characteristics are shown in Fig. 3. There were significant differences in the survival rate between patients with values above and below the cut-offs for intraoperative blood loss, operation time, and the number of positive lymph nodes. However, no significant differences in the survival rate were noted between patients who underwent different remnant gastrectomy procedures or between those with values above and below the cut-off for the number of retrieved lymph nodes.

### Multivariate analysis of prognostic factors

Multivariate analysis revealed that the T stage (hazard ratio, 5.593; 95% confidence interval [CI], 1.183–26.452; *p* = 0.030) and venous invasion (hazard ratio, 3.351; 95% CI, 1.030–10.903; *p* = 0.045) were significant independent risk factors for the long-term survival of patients who underwent radical resection for RGC (Table 3).

**Table 3:** Multivariate analysis of prognostic factors using the Cox proportional hazard model

Prognostic factors	HR	95% CI Lower limit	95% CI Upper limit	***P***
T factor (T2–T4/T1)	5.593	1.183	26.452	**0.030**
N factor (N+/N−)	2.535	0.643	9.991	0.184
Ly factor (Ly+/Ly−)	0.364	0.098	1.361	0.133
V factor (V+/V−)	3.351	1.030	10.903	**0.045**
Histological type (tub1, tub2/por, sig, others)	0.481	0.184	1.258	0.136
Tumor size (> 34 mm/< 34 mm)	2.499	0.627	9.960	0.194
The number of positive lymph nodes (> 1/≦1)	0.481	0.133	1.739	0.265

*CI*, confidence interval; *HR*, hazard ratio

## Discussion

The survival rate of patients with gastric cancer has improved due to early detection and treatment [10, 11]. As a result, more patients may develop RGC [5]. Previous studies have indicated that the pattern of reconstruction is associated with the incidence and location of the RGC [6–8, 12, 13]. However, there have been few reports on the long-term prognosis and associated clinicopathological factors of remnant gastrectomy, and its management remains controversial. Therefore, in this study, we examined the clinicopathological factors associated with the long-term outcomes of RGC.

Our findings showed that the pathological T stage and venous invasion were significant independent risk factors for survival among patients with RGC; however, the pathological N stage was not significantly associated with long-term survival. Several studies have suggested that endoscopic surveillance is crucial, because early detection of RGC leads to a better prognosis [5, 6, 8, 14]. Thus, given the prognostic advantage of detecting RGC at an early T stage, follow-up endoscopy after distal gastrectomy is recommended for all patients. However, there is no consensus on the extent of lymphadenectomy during surgery for RGC. Since a lymph node dissection was performed in a previous surgery, the N factor in our current study might not be associated with the prognosis for RGC. However, in the current study, we found that venous invasion was an independent risk factor, along with the T stage. Nishibeppu et al. showed that venous invasion was a risk factor for recurrence after gastrectomy followed by adjuvant chemotherapy for stage III gastric cancer [15]. Another study showed that venous and nerve invasion were prognostic factors of postoperative survival in patients with resectable cancer of the rectum [16]. Based on these results, including our analysis, we believed that venous invasion could be an important risk factor for prognosis. Thus, we recommend that RGC with venous invasion should be specifically targeted to improve the prognosis of patients and suggest the possibility of it serving as an indication for more intensive treatment, such as adjuvant chemotherapy. Some studies have reported that early diagnosis and curative resection are important to improve prognosis [17, 18]; however, there have been no studies on the association of venous invasion with worse prognosis. Therefore, evaluating venous invasion may help determine therapeutic strategies, including adjuvant chemotherapy, for RGC.

In this study, we did not test for *Helicobacter pylori* (HP) infection in the RGC patients. A previous study suggested that prophylactic HP eradication after endoscopic resection of early gastric cancer might be effective for preventing the development of metachronous gastric carcinoma [19]. However, the importance of HP eradication after gastrectomy remains unclear because some researchers reported that a positive HP test may be an independent risk factor for cancer recurrence [20], while other researchers indicated that HP eradication in patients with GC who underwent distal gastrectomy did not contribute to long-term postoperative survival [21]. Thus, we considered that performing follow-up endoscopy was more crucial than eradicating HP, due to the high number of elderly patients with RGC.

This study had some limitations. First, this study was a single institutional retrospective study with a relatively small sample size and limited clinical analysis of HP infection, due to our inability to obtain detailed data. Recently, some studies have been conducted regarding HP infection after gastrectomy. In the future, we should determine the relationship between RGC and HP infection, using accumulated prospective data.

Second, the number of patients in each category was unequal: the number of patients with initial malignant tumors was higher than that of patients with initial benign tumors. However, this difference may be attributable to the recent trend of prolonged survival in patients with cancer.

## Conclusions

Although some patients had lymph node metastasis, the important prognostic factors for long-term survival of patients with RGC were the T stage and venous invasion. Therefore, they may be keys to managing and identifying therapeutic strategies for RGC to achieve better prognosis.

## Supplementary Information

**Additional file 1: Supplemental Fig. 1.** Long-term survival outcomes based on diet, history of alcohol drinking, and history of smoking.

**Additional file 2: Supplemental Fig. 2.** Long-term survival outcomes based on pathological T stages: (A) pathological T1 and pT2, (B) pT1 and pT3, (C) pT1and pT4, (D) pT2 and pT3, (E) pT2 and pT4, and (F) pT3 and pT4.

## Data Availability

The datasets used during the current study are available from the corresponding author on reasonable request.

## Abbreviations

BMI: Body mass index
CT: Computed tomography
EGD: Esophagogastroduodenoscopy
HP: *Helicobacter pylori*
RGC: Remnant gastric cancer
