# Supplementary figures and images for: T stage and venous invasion are crucial prognostic factors for long-term survival of patients with remnant gastric cancer: a cohort study
Source: World J Surg Oncol. 2021 Sep 27;19:291. doi: 10.1186/s12957-021-02400-5 (PMC8477455; doi:10.1186/s12957-021-02400-5)

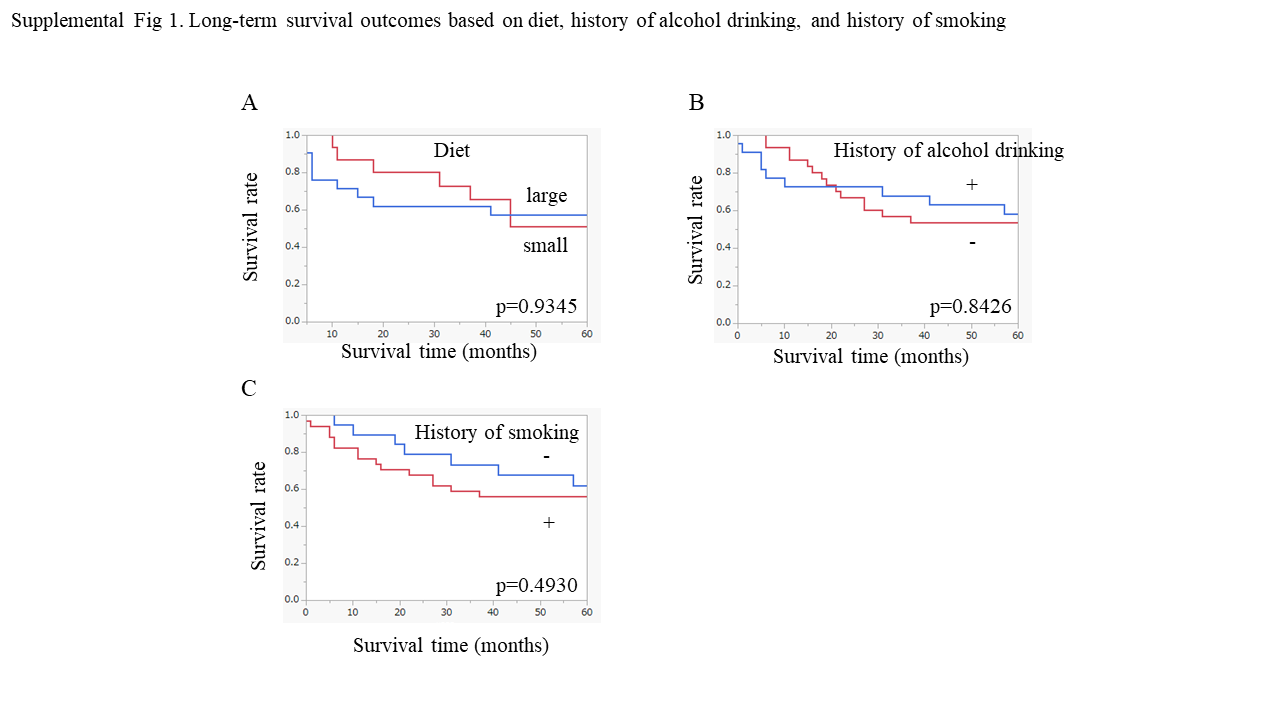

Supplement: Supplementary file 1 — Additional file 1: Supplemental Fig. 1. Long-term survival outcomes based on diet, history of alcohol drinking, and history of smoking. [file 12957_2021_2400_MOESM1_ESM.tif]

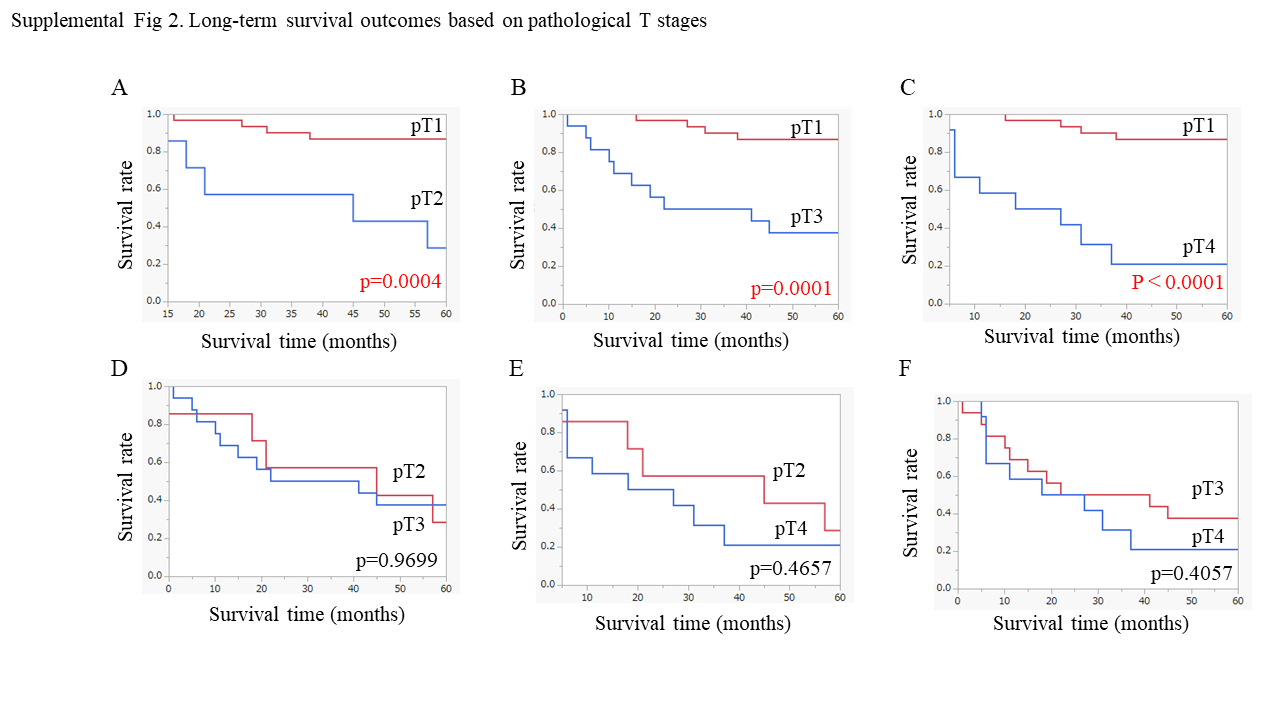

Supplement: Supplementary file 2 — Additional file 2: Supplemental Fig. 2. Long-term survival outcomes based on pathological T stages: (A) pathological T1 and pT2, (B) pT1 and pT3, (C) pT1and pT4, (D) pT2 and pT3, (E) pT2 and pT4, and (F) pT3 and pT4. [file 12957_2021_2400_MOESM2_ESM.tif]
